# Differences in Characteristics between Older Adults Meeting Criteria for Sarcopenia and Possible Sarcopenia: From Research to Primary Care

**DOI:** 10.3390/ijerph19074312

**Published:** 2022-04-04

**Authors:** Hyung Eun Shin, Miji Kim, Chang Won Won

**Affiliations:** 1 Department of Biomedical Science and Technology, Graduate School, Kyung Hee University, Seoul 02447, Korea; she9310@gmail.com; 2 Department of Biomedical Science and Technology, College of Medicine, East-West Medical Research Institute, Kyung Hee University, Seoul 02447, Korea; 3 Elderly Frailty Research Center, Department of Family Medicine, College of Medicine, Kyung Hee University, Seoul 02447, Korea

**Keywords:** assessment, community-dwelling, primary care, sarcopenia, sex difference

## Abstract

Identification of possible sarcopenia, which is a simple assessment of sarcopenia, has been proposed for the earlier detection of sarcopenia in primary care settings; however, there are no studies comparing the differences in characteristics of older adults with possible sarcopenia or sarcopenia. This study aimed to compare the characteristics of “possible sarcopenia” in real-world primary care and “sarcopenia” in research settings. A total of 2129 older adults were enrolled from the Korean Frailty and Aging Cohort Study. Possible sarcopenia and sarcopenia were defined using Asian Working Group for Sarcopenia 2019; the possible sarcopenia for real-world primary care was defined by a combination of case findings using low calf circumference or the SARC-F questionnaire and 5-times chair stand test, without considering the measurement of handgrip strength. The prevalence of possible sarcopenia was higher in women than in men; however, that of sarcopenia was higher in men than in women (all, *p* < 0.001). Older men and women with possible sarcopenia had a lower education level, longer time taken for the Timed Up and Go test, more severe mobility limitation, lower scores on the EuroQol-5 dimension and 12-item short-form survey for physical health, and more cognitive dysfunction than those with sarcopenia did (all, *p* < 0.05). In conclusion, the participants with possible sarcopenia differed from those with sarcopenia in some characteristics. Identifying differences in characteristics may be helpful to screening and earlier diagnosis of sarcopenia in real-world primary care, as well as in research, which can lay the foundations for personalized lifestyle intervention in diet and exercise.

## 1. Introduction

Sarcopenia is defined as the age-related loss of skeletal muscle mass, muscle strength, and/or physical performance, leading to adverse health outcomes such as functional decline, disability, falls, morbidity, and mortality in older adults [1,2]. The presence of sarcopenia increases the risk of hospitalization and related healthcare expenditures [3]. More importantly, sarcopenia has characteristics in common with physical frailty, and it has been suggested that sarcopenia constitutes the substrate of frailty [4,5]. Indeed, the adverse health outcomes of frailty can be affected by sarcopenia [6]. Considering the relationship between sarcopenia and physical frailty, the earlier detection of sarcopenia can help prevent progression to physical frailty. As of October 2016, sarcopenia was assigned as an independent medical condition (M62.84) by the new International Classification of Disease, Tenth Revision, Clinical Modification (ICM-10-CM) [7]. It stimulated the diagnosis of sarcopenia at an early stage and its managements for physicians. Moreover, in 2019, Asian Working Group for Sarcopenia (AWGS) introduced “possible sarcopenia” specifically for use in primary healthcare to enable earlier lifestyle interventions.

The consensus diagnostic criteria for sarcopenia were presented by AWGS 2019 [2]. To diagnose definitive sarcopenia according to the AWGS 2019, several validated tools are needed to assess skeletal muscle mass, muscle strength, and physical performance. The most recommended and validated measurement is dual-energy X-ray absorptiometry (DXA) for skeletal muscle mass, a handgrip dynamometer for muscle strength, and 6 m gait speed for physical performance [8]. These tools have been used to diagnose definitive sarcopenia in well-equipped facilities, such as hospital or research settings. However, such tools and room space are not always available in primary care settings, due to high cost and lack of space [8].

To promote the earlier detection of sarcopenia in primary care settings, AWGS 2019 introduced a simple assessment to define “possible sarcopenia” in settings without advanced diagnostic equipment [1]. Lifestyle intervention in diet and exercise is recommended for those who have been detected early in primary care settings [1]. According to the AWGS 2019 algorithm, calf circumference (CC), a simple five-item questionnaire (SARC-F), or the SARC-F combined with CC (SARC-CalF) is used for case-finding. After case-finding, possible sarcopenia was defined using the assessment of handgrip strength or 5-times chair stand test (5-CST) [1]. Based on the AWGS 2019 algorithm, we operationally defined “possible sarcopenia” for real-world primary care settings as a combination of case-finding with CC or the SARC-F questionnaire and assessment of physical performance using 5-CST without considering the measurement of handgrip strength. This is because measuring handgrip strength requires a dynamometer for strength of grip, but most primary care settings are not equipped with it. Thus, in primary care settings, simple measures composed of a questionnaire, anthropometric measurements, and 5-CST can be appropriate alternatives to highly specialized tools.

Ueshima et al., (2021) and Miura et al., (2021) described the characteristics of possible sarcopenia. In particular, Ueshima et al. defined possible sarcopenia using the CC, handgrip strength, and repeated 5-CST, and Miura et al. defined possible sarcopenia using only the CC and handgrip strength. Both studies compared the characteristics of possible sarcopenia with those of nonsarcopenia [9,10]; however, to date, no study has compared between older adults with possible sarcopenia and those with definitive sarcopenia. Understanding the characteristics of older adults with “possible sarcopenia” in primary care settings compared with those with “sarcopenia” in hospital or research settings would provide an opportunity to explore the comparability and limitations of using possible sarcopenia in real-world primary care settings. Therefore, the purpose of our study was to compare the characteristics of older adults with possible sarcopenia in real-world primary care provided with a handgrip dynamometer and those with definitive sarcopenia.

## 2. Materials and Methods

### 2.1. Participants

The Korean Frailty and Aging Cohort Study (KFACS) is an ongoing longitudinal study, and its baseline survey was conducted from May 2016 to November 2017. The participants were recruited from age- and sex-stratified community-dwelling residents in ten centers in urban, suburban, and rural areas. The ratio of participants in the 70–74, 75–79, and 80–84 age groups was 6:5:4, and the ratio of men to women was 1:1 [11]. Of the 3011 participants enrolled at baseline, 2129 participants were included in cross-sectional analysis after applying certain exclusion criteria; those who underwent bioelectrical impedance analysis (BIA) for body composition were excluded because of the systematic bias between BIA and DXA in measuring appendicular skeletal muscle mass (*n* = 610) [12], and those who had artificial joints, pins, plates, metal suture materials, or other types of metal objects in the appendicular body regions were excluded (*n* = 272). The KFACS protocol was approved by the Clinical Research Ethics Committee of Kyung Hee University Hospital (Institutional Review Board [IRB] number: 2015-12-103). This study used the KFACS dataset and was exempted from IRB approval from the Clinical Research Ethics Committee of the Kyung Hee University Hospital (IRB number: 2021-10-020).

### 2.2. Definition of “Possible Sarcopenia” for Real-World Primary Care Settings and “Sarcopenia”

Based on the AWGS 2019 guidelines, “possible sarcopenia” for real-world primary care settings was defined by a combination of case findings using the low CC (<34 cm in men and <33 cm in women) or the SARC-F questionnaire (score ≥ 4) and 5-CST (≥ 12 s for both sexes), without considering the measurement of handgrip strength. The definitive diagnosis of “sarcopenia” was defined as low muscle mass (<7.0 kg/m^2^ for men and <5.4 kg/m^2^ for women) and low handgrip strength (<28 kg for men and <18 kg for women) and/or low physical performance (<1.0 m/s for 6 m gait speed or ≥12 s for 5-CST or ≤9 for SPPB).

### 2.3. Diagnostic Measures for Possible Sarcopenia and Sarcopenia

The SARC-F questionnaire assessed components covering strength, assistance with walking, rising from a chair, climbing stairs, and falling. The Korean version of the questionnaire consists of five components, each of which is scored from 0 to 2, with a total possible score of 0 to 10. A total score of four or higher was considered possible sarcopenia [13,14]. Calf circumference of the left leg was measured in the standing position. Measurements were made at the point of the maximum circumference without compressing the subcutaneous tissue. The 5-CST measured the time that participants took to stand up from and sit down on a standardized chair five times as quickly as possible while crossing both arms on the chest. The time taken to perform five trials was measured.

Appendicular skeletal muscle mass (ASM) was measured using DXA (Hologic DXA; Hologic Inc., Bedford, MA, USA and Lunar GE 156 Healthcare, Madison, WI, USA). The segmental lean masses of both arms and legs were summed to obtain the ASM. The ASM index was calculated using the formula ASM/(height)^2^. The handgrip strength of each hand was measured twice using a digital handgrip dynamometer (T.K.K.5401; Takei Scientific 160 Instruments Co., Ltd., Tokyo, Japan), and the maximum value was used as the output. Usual gait speed was assessed over 4 m using an automatic timer (Gait speed meter, Dynamicphysiology, Daejeon, Korea), with acceleration and deceleration phases of 1.5 m each. It was measured twice, and the mean of the two trials was used. The short physical performance battery (SPPB) test assesses lower extremity function using usual gait speed, balance (tandem, semi-tandem, and side-by-side stands), and the 5-times chair stand test (5-CST) measures. Participants were assigned a score from 0 to 4 in each test, with a total possible score of 0 to 12. The Timed Up and Go (TUG) test assesses functional mobility. In the TUG test, participants were asked to stand up from an armchair of a standard height, walk a 3 m distance with their own comfortable and safe walking pace, turn around, return to the chair, and sit down. The time taken from standing up to sitting down was measured.

### 2.4. Other Clinical Characteristics

All information was obtained through face-to-face interviews conducted by experienced investigators. Sociodemographic and lifestyle information on age, education level, marital status, social security recipient, current worker, physical activity level, and malnutrition were collected using a standardized questionnaire. Body mass index (BMI) was calculated using the formula weight (kg)/height squared (m^2^). Low physical activity level was determined using the International Physical Activity Questionnaire and defined as a total energy expenditure of <494.65 kcal for men and <283.50 kcal for women. These values correspond to the lowest 20% of the total energy consumed by population-based older adults in South Korea [15]. Nutritional intake was assessed using the Korean version of the Mini-Nutritional Assessment Short Form (MNA-SF), and malnutrition was defined as an MNA-SF score of <11 [16]. Surveys on hospitalization in the past year, chewing ability, and self-reported medical status were conducted.

Comorbidity status was defined as having two or more of the following diseases: hypertension, diabetes mellitus, dyslipidemia, myocardial infarction, congestive heart failure, angina pectoris, cerebrovascular disease, peripheral vascular disease, osteoporosis, osteoarthritis, rheumatoid arthritis, asthma, or chronic obstructive pulmonary disease. All diseases were self-reported as diagnosed by the physicians. Polypharmacy was defined as taking at least five medications concurrently. The definition of hearing and visual impairment was a minimum pure-tone average value of ≥40 dB for better hearing and maximum visual acuity of <0.3, respectively. ADL disability was defined as dependence on others for at least one of the following seven domains: bathing, continence, dressing, eating, transfer, and washing the face and hands. IADL disability was defined as dependence on others for two or more of the following ten domains: decorating, housework, preparing meals, laundry, short outings, use of transportation, shopping, handling money, using the telephone, and taking medicines. Falls in the past year, defined as any fall event within the last year, were recorded. Severe mobility limitation was defined as cases where the participants responded “very difficult” or “impossible” to at least one of the following questions: “How difficult is it for you to climb ten stairs without rest?” or “How difficult is it for you to walk about 400 m without rest?”. QoL was determined using the EuroQol 5-dimension scale (EQ-5D), EuroQol Visual Analog Scale (EQ-VAS), and 12-item short-form health survey (SF-12). Depressive symptoms were assessed using the Korean version of the short-form Geriatric Depression Scale (SGDS-K), and a score of ≥6 was considered depression [17]. Cognitive dysfunction was diagnosed if participants had a score of <24 in the Korean version of the Mini-Mental State Examination (MMSE-KC) [18].

Social support was assessed using the Enhancing Recovery in Coronary Artery Disease Social Support Instrument. An individual was considered as having poor social capital if they responded “no” to the following question: “Are you able to participate in any social gathering such as social, religious, cultural, sports leisure, civic, political, volunteer, and learning organization?”. Social networks were assessed using the Practitioner Assessment of Network Type Instrument. Interactions with family, friends, and neighbors were categorized as “high” (every day, 2–3 days/week, or ≥1 day/week) or “low” (≤1 day/month).

### 2.5. Statistical Analysis

Data are presented as means (± standard deviations (SDs)) or frequencies (percentages). The generalized estimating equation (GEE) model was used to compare the characteristics of participants without sarcopenia (no sarcopenia), with possible sarcopenia, and with sarcopenia due to overlapping participants in the possible sarcopenia and sarcopenia groups. Post hoc tests were conducted using an independent t-test for continuous variables and chi-square or Fisher’s exact test for categorical variables (no sarcopenia vs. possible sarcopenia or no sarcopenia vs. sarcopenia) with the GEE model (possible sarcopenia vs. sarcopenia).

The GEE was used to estimate the odds ratios and 95% confidence intervals (CIs) for the predictors of possible sarcopenia compared with sarcopenia. Model 1 included sociodemographic and lifestyle factors. Model 2 included anthropometric and physical performance variables in addition to sociodemographic and lifestyle factors. Model 3 further included medical and health status, and further psychological, cognitive, and social factors were included in Model 4. Variables with a *p*-value of <0.1 in the univariate analysis were entered into the GEE model. A two-sided *p*-value of <0.05 was considered statistically significant. The statistical analyses were performed using SPSS (version 26.0; IBM Corp., Armonk, NY, USA).

## 3. Results

**Figure 1:**
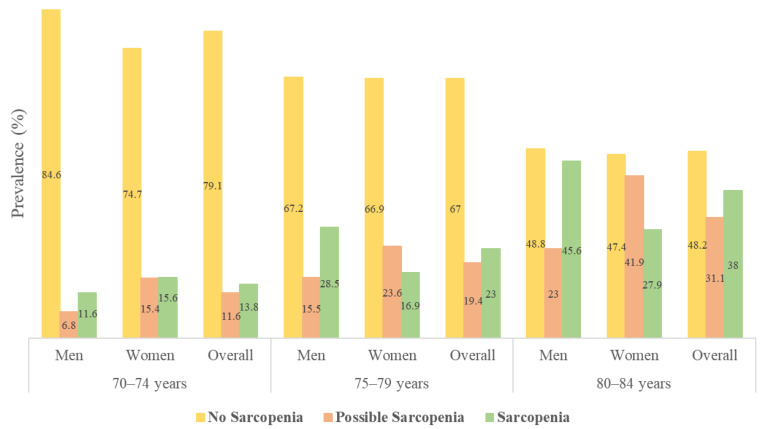
Prevalence (%) of no sarcopenia, possible sarcopenia, and sarcopenia. The prevalence of possible sarcopenia and sarcopenia, as defined by the Asian Working Group for Sarcopenia 2019, was significantly different between men and women (*p* < 0.001). Possible sarcopenia was defined as either the calf circumference or a simple five-item questionnaire (SARC-F) and the 5-times chair stand test. Sarcopenia was defined as low muscle mass, plus low handgrip strength, and/or low physical performance.

**Figure 2:**
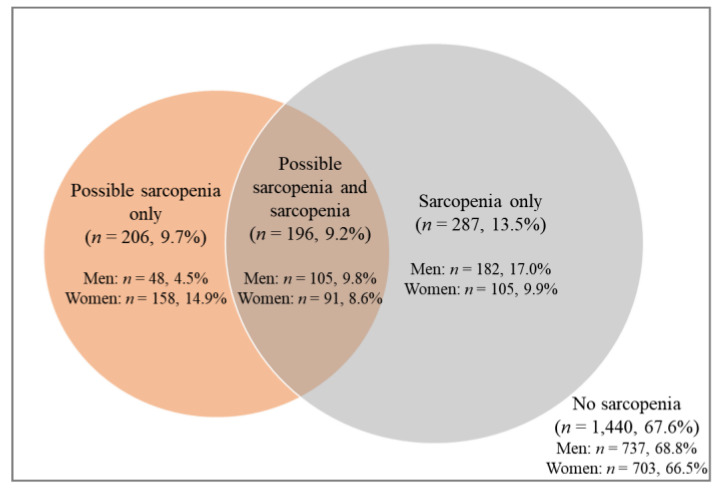
Venn diagram depicting the overlaps between possible sarcopenia and sarcopenia. The number of participants with both possible sarcopenia and sarcopenia was 196 (9.2%); 105 (9.8%) were men and 91 (8.6%) were women.

The prevalence of no sarcopenia, possible sarcopenia, and sarcopenia was 67.6% (*n* = 1440), 18.9% (*n* = 402), and 22.7% (*n* = 483), respectively. The prevalence of possible sarcopenia and sarcopenia significantly increased with age, from 11.6% and 13.8%, respectively, in the 70–74 age group to 31.1% and 38.0%, respectively, in the 80–84 age group. The prevalence of possible sarcopenia was significantly higher in women than in men regardless of age group (*p* < 0.01), but that of sarcopenia was significantly higher in men than in women in the 75–79 (*p* < 0.001) and 80–84 (*p* < 0.001) age groups except the 70–74 age group (*p* = 0.085), as shown in Figure 1. Overlaps of possible sarcopenia and sarcopenia are shown in Figure 2. The prevalence of overlapping participants of possible sarcopenia and sarcopenia was 9.2% (*n* = 196).

Table 1 shows the comparisons of the characteristics of possible sarcopenia and sarcopenia; the possible sarcopenia group had a lower education, longer times taken in the Timed Up and Go (TUG) test, more severe mobility limitations, lower EuroQol-5 dimension (EQ-5D) score, lower 12-item short-form survey (SF-12) physical health scores, and more cognitive dysfunction in both men and women. Women with possible sarcopenia were more likely to be unmarried and had a higher body mass index (BMI), greater waist circumference, higher prevalence of instrumental activities of daily living (IADL) disability, and less osteoporosis than those with sarcopenia; however, these differences were not found in men. Men with possible sarcopenia had a lower prevalence of hearing impairment and lower social network with neighbors than those with sarcopenia.

In Table 2, multivariate analysis was performed to identify the predictors of possible sarcopenia compared with those of sarcopenia in men. In GEE analysis adjusted for confounding factors (Model 4), lower education (odds ratio (OR) = 1.78, 95% CI = 1.09–2.90), longer times taken in TUG test (OR = 1.09, 95% CI = 1.00–1.19), no hearing impairment (OR = 0.54, 95% CI = 0.32–0.93), and higher SF-12 mental health scores (OR = 1.04, 95% CI = 1.00–1.07) were more likely to occur in possible sarcopenia than in sarcopenia in men.

The predictors of possible sarcopenia compared with sarcopenia in women are presented in Table 3. After adjusting for confounders (Model 4), lower education was at 1.91 times higher odds of being possibly sarcopenic (OR = 1.91, 95% CI = 1.17–3.14). A higher prevalence of IADL disability was more likely to occur in women with possible sarcopenia than those with sarcopenia after adjusting for all covariates (OR = 4.61, 95% CI = 1.21–17.50).

## 4. Discussion

In this cross-sectional study of community-dwelling older adults aged over 70 years, we compared the characteristics of older adults with “possible sarcopenia” in real world primary care settings with those with “sarcopenia” in research using the AWGS 2019 criteria. We found that older men and women with possible sarcopenia had a lower education level, longer times taken in the TUG test, more common severe mobility limitations, lower EQ-5D index and SF-12 physical health scores, and more cognitive dysfunction than those with sarcopenia did. We also found that age, marital status, BMI, waist circumference, IADL disability, and osteoporosis differed between possible sarcopenia and sarcopenia in women. The prevalence of hearing impairment was lower in men with possible sarcopenia than in those with sarcopenia. Identification of the different characteristics of possible sarcopenia and sarcopenia is meaningful in that it enables earlier detection and lifestyle interventions that can help delay or reverse the symptoms.

Those with lower education levels tend to engage in strenuous physical work at their workplace or vigorous household chores. The participants with more than 12 years of education were less likely to engage in strenuous work-related activities than those with less than 8 years of education were [19]. Strenuous work-related physical activities may increase the risk of mobility limitation [20]. Thus, it can be speculated that work-related physical activities may explain the relations of low education with severe mobility limitation. In addition, the culprit for the higher prevalence of severe mobility limitation in possible sarcopenia than in sarcopenia might be SARC-F. Indeed, a previous study revealed that SARC-F detects more participants with mobility limitations as well as sarcopenia [21]. It has been reported that severe mobility limitation negatively affects the quality of life and cognitive dysfunction in older adults. Several studies have reported that mobility limitations may decrease the SF-12 [22] and EQ-5D scores in older adults [23]. Tolea et al. reported an association between mobility limitation and cognitive function, which follows a dose–response pattern and operates bidirectionally, suggesting that the early stage of mobility limitation can predict cognitive dysfunction [24].

The TUG tests are reliable and valid clinical tools that can easily measure lower extremity function [25]. In our study, the 5-CST, which measures lower extremity function, was necessarily used to define possible sarcopenia, but optionally used to define definitive sarcopenia. Some older adults with low 5-CST may not be included in the definitive sarcopenia group. Indeed, the time taken to complete the 5-CST in older adults with possible sarcopenia was higher than in those with sarcopenia (14.42 s vs. 12.50 s in men and 15.39 s vs. 13.72 s in women). This may be attributed to a higher number of older adults with decreased lower extremity function, assessed by the 5-CST, being included in the possible sarcopenia group than in the sarcopenia group. With the same reason, the TUG test results could be worse in older adults with possible sarcopenia. In our study, we observed the differences in the time taken to complete the 5-CST by sex. Several studies have suggested reference values of 10–17 s to predict the adverse health outcomes in older adults [26,27,28,29], with one study reporting age-, sex-, and age-and-sex-stratified cutoff values against a higher risk of functional decline in older adults [27]. However, the 5-CST cutoff values stratified by age and sex have not been identified to date. In particular, it is necessary to identify the optimal cutoff values for men and women based on differences in muscle mass and muscle strength.

Older women with possible sarcopenia had significantly higher BMIs and waist circumferences than those with sarcopenia. This may be because all women with sarcopenia met the criteria for low muscle mass, but those with possible sarcopenia did not. Furthermore, older women with possible sarcopenia in our study had a mean waist circumference of 85.4 cm, which exceeded the cut-off point of waist circumference for abdominal obesity in Korean women [30]. This indicates that women with possible sarcopenia in this study were more likely to have abdominal obesity, which is a significant risk factor for IADL disability [31]. Thus, it can be explained that the prevalence of IADL disability was higher in women with possible sarcopenia than in those with sarcopenia, as women with possible sarcopenia are more abdominally obese than those with sarcopenia.

Older men with possible sarcopenia had a lower prevalence of hearing impairment than those with sarcopenia. In a previous study, low muscle mass was significantly associated with a higher level of hearing loss, and the pathophysiology of this association could be related to reduced blood flow [32]. A greater volume of muscle mass requires more blood supply, leading to higher cardiac output, stroke volume, and arterial size adaptation. In addition, men with sarcopenia tend to have greater arterial stiffness [33]. This dysfunction in vascular mechanics may also interrupt blood flow to the cochlea and decrease cochlear function, which can lead to a poor hearing threshold.

After adjusting for confounders, lower education, longer time taken for TUG test, no hearing impairment, and higher SF-12 mental health in men, and lower education and more IADL disability in women were found to be significant predictors of possible sarcopenia compared with sarcopenia. The higher SF-12 mental health scores in older men with possible sarcopenia may have been influenced by adjustments of the education level. In a previous study, individuals with a high education level had higher SF-12 mental health scores than those with a low education level [34]. In our study, the SF-12 mental health scores showed no difference between possible sarcopenia and sarcopenia in univariate analysis, whereas the odds ratio for the scores became higher in possible sarcopenia in multivariate analysis including the education level.

We compared the characteristics of “possible sarcopenia” in real-world primary care settings and definitive “sarcopenia” in hospital or research settings using AWGS 2019 criteria. Several operational definitions of sarcopenia have been proposed. The clinical definition and consensus diagnostic criteria for sarcopenia were developed by the European Working Group on Sarcopenia in Older People, International Working Group on Sarcopenia, Foundation for the National Institutes of Health Sarcopenia Project, and AWGS, depending on the different populations [1,35,36,37]. Our previous work showed that the prevalence of sarcopenia was distributed from 8.4% to 25.5% for men and from 4.7% to 16.2% for women according to diagnostic criteria for sarcopenia [38]. Therefore, we defined the possible sarcopenia and sarcopenia using the AWGS 2019 criteria; otherwise, if different criteria for possible sarcopenia and sarcopenia were applied, the results might be different.

In our study, possible sarcopenia prevalence was significantly higher in women than in men, whereas sarcopenia prevalence was higher in men than in women. A previous study reported that women were more likely to have knee osteoarthritis than men, especially after menopause due to estrogen deficiency [39,40], and those with knee osteoarthritis may have poor physical performance in the chair stand test, which was the main component of possible sarcopenia in this study [41]. The chair stand task involves greater joint torque and sufficient joint range of motion to perform it completely. Knee osteoarthritis is one of the diseases that can negatively affect joint torque and range of motion, which could make the chair stand task performed poorly [42]. Indeed, women had a higher osteoarthritis prevalence than men in our study (W: 29.9%, M: 11.6%, *p* < 0.001), and the mean 5-CST time was 14.6 s for women and was 13.2 s for men, taking longer for women to complete the task. Additionally, it may also be explained by finding that lower extremity strengths are lower in women than men [43]. This explanation is possible because the 5-CST is known as a surrogate for lower extremity strength, although the test is regarded as a surrogate for physical performance in AWGS 2019. Furthermore, sex differences in assessing CC and SARC-F used for case-finding of possible sarcopenia may have influenced the higher prevalence of possible sarcopenia in women. In our study, the prevalence of low CC was higher in women than in men (50.0% vs. 37.2%), and that of a SARC-F score of ≥4 was also higher in women than in men (11.9% vs. 3.2%).

In addition, several pathological factors such as aging, hormonal changes, and inflammation could be responsible for this difference [44]. The reason for this is not yet clear, but it could be attributed to hormone changes with age, such as testosterone levels. Testosterone has direct anabolic effects on skeletal muscles and increases muscle mass and strength. For women, free testosterone levels have no apparent relationship with age; however, free testosterone levels gradually decrease with age in men, which may affect the decrease in muscle mass and strength [45,46]. Thus, age-related decreases in testosterone levels may have influenced the higher prevalence of sarcopenia, especially in men.

Meanwhile, a large proportion of participants with sarcopenia would not have been detected using only an assessment for possible sarcopenia. The reason for this could be an assessment for possible sarcopenia, which consisted of proxy indicators. A recent study revealed that SARC-F identified 67.5% of patients at risk for sarcopenia with fair sensitivity (67–74%) and poor specificity (32–37%) [47]. Calf circumference has an accuracy of 77% for men (sensitivity, 85%; specificity, 66%) and 75% for women (sensitivity, 91%; specificity, 28%), although it is a valid proxy for appendicular skeletal muscle mass [48]. Due to this gap, some participants with definitive sarcopenia may not be included in the possible sarcopenia.

We defined “possible sarcopenia” to reflect real-world primary care. The lack of highly specialized equipment to measure muscle mass and sufficient space to assess physical performance are challenges in primary care. To overcome these practical difficulties, possible sarcopenia could be adopted in primary care, and therefore, it is necessary to identify whether those diagnosed with possible sarcopenia are more comparable to those with definitive sarcopenia. Ueshima et al. showed the high diagnostic accuracy of “possible sarcopenia” against definitive sarcopenia using AWGS 2019 [10]. In this study, only CC was used for case-finding of possible sarcopenia [10], whereas we used not only CC but also SARC-F questionnaires for case-finding of possible sarcopenia. In addition, Ueshima et al. defined sarcopenia by using the algorithm including case-finding of CC suggested by AWGS 2019, whereas we applied classical diagnostic criteria of sarcopenia in hospital or research settings using AWGS 2019 guidelines, not considering case-finding.

We included the 5-CST only for muscle strength to assess possible sarcopenia and not handgrip strength in this study. Although the 5-CST has been suggested as a feasible tool for assessing physical performance in AWGS 2019, it can also be used as a valid and reliable alternative for assessing muscle strength when handgrip dynamometers are unavailable [8,49]. The test has been proven to be practical for evaluating muscle strength as well as physical performance in primary care settings [50]. Several studies have shown that the 5-CST is significantly associated with muscle strength [51] and walking speed [52], and is also a significant predictor of subsequent falls and disability in older adults [53]. Meanwhile, definitive sarcopenia was defined as low muscle mass, plus low handgrip strength, and/or low physical performance according to AWGS 2019; gait speed was recommended for physical performance by AWGS guidelines in 2014, and the SPPB test and 5-CST have been additionally proposed as surrogates for gait speed in sarcopenia diagnosis by AWGS 2019 guidelines. Hence, low physical performance was defined based on at least one of the following three criteria: the SPPB test, gait speed, and 5-CST.

Our study had several limitations. First, we used the diagnostic criteria of AWGS 2019 to diagnose sarcopenia; therefore, they cannot be applied to non-Asians. Second, the participants in our study were ambulatory community-dwelling older adults. Therefore, the results may not be generalizable to other populations and settings.

## 5. Conclusions

In our study, the participants with possible sarcopenia differed from those with sarcopenia in some characteristics. Older men and women with possible sarcopenia had a lower education level, lower functional ability, lower quality of life, and more cognitive dysfunction than those with sarcopenia. Identification of the different characteristics of older adults with possible sarcopenia or sarcopenia in Asians is meaningful in that it allows us to prejudge and prepare for the strengths and limitations of diagnosing and managing “possible sarcopenia” with lifestyle interventions in real world primary care. Moreover, these findings are expected to help provide lifestyle interventions optimized for the characteristics of the patients with possible sarcopenia.

## Figures and Tables

**Table 1:** Characteristics of the study participants in the no sarcopenia, possible sarcopenia, and sarcopenia groups.

Variable	Men				Women			
	No Sarcopenia *n* = 737	Possible Sarcopenia *n* = 153	Sarcopenia *n* = 287	*p*-Value	No Sarcopenia *n* = 703	Possible Sarcopenia *n* = 249	Sarcopenia *n* = 196	*p*-Value
*Sociodemographic and lifestyle factors*								
Age, years	75.5 ± 3.7	78.4 ± 3.7 ^†^	78.5 ± 3.7 ^‡^	<0.001	74.9 ± 3.7	77.1 ± 4.0 ^†^	76.2 ± 4.0 ^‡,^*	<0.001
Low education level, <7 years	141 (19.1)	58 (38.2) ^†^	67 (23.3) *	0.007	334 (47.6)	176 (70.7) ^†^	95 (48.5) *	0.001
Marital status (without partner)	66 (9.0)	21 (13.7)	37 (12.9)	0.034	346 (49.2)	153 (61.4) ^†^	95 (48.5) *	0.149
Social security recipient	42 (5.7)	8 (5.3)	17 (6.0)	0.935	56 (8.0)	21 (8.6)	15 (7.7)	0.978
Current worker	203 (27.6)	29 (19.0) ^†^	59 (20.6) ^‡^	0.006	163 (23.2)	52 (21.0)	38 (19.4)	0.223
Current smoker	69 (9.4)	24 (15.7) ^†^	42 (14.6) ^‡^	0.007	5 (0.7)	6 (2.4) ^†^	5 (2.6) ^‡^	0.026
Alcohol intake (≥2 times/week)	253 (34.3)	50 (32.7)	90 (31.4)	0.356	23 (3.3)	12 (4.8)	5 (2.6)	0.873
Low physical activity	54 (7.4)	33 (22.8) ^†^	48 (16.9) ^‡^	<0.001	44 (6.3)	55 (22.6) ^†^	30 (15.3) ^‡^	<0.001
*Anthropometric measurements and physical performance*								
BMI, kg/m^2^	24.5 ± 2.8	22.4 ± 2.9 ^†^	22.5 ± 2.6 ^‡^	<0.001	24.9 ± 2.7	23.7 ± 3.1 ^†^	22.8 ± 2.6 ^‡,^*	<0.001
Waist circumference, cm	89.3 ± 8.1	85.9 ± 9.0 ^†^	86.6 ± 8.9 ^‡^	<0.001	86.7 ± 7.9	85.4 ± 9.0 ^†^	82.7 ± 7.8 ^‡,^*	<0.001
ASM index, kg/m^2^	7.3 ± 0.8	6.6 ± 0.9 ^†^	6.3 ± 0.5 ^‡,^ *	<0.001	6.0 ± 0.7	5.7 ± 0.7 ^†^	5.0 ± 0.3 ^‡,^ *	<0.001
Timed Up and Go test, s	9.5 ± 1.8	12.4 ± 3.6 ^†^	11.4 ± 2.7 ^‡,^*	<0.001	9.8 ± 1.9	12.6 ± 3.6 ^†^	11.1 ± 2.5 ^‡,^ *	<0.001
*Health status*								
Comorbidities (≥2 diseases)	304 (41.2)	64 (41.8)	121 (42.2)	0.782	398 (56.6)	150 (60.2)	128 (65.3) ^‡^	0.770
Polypharmacy (≥5 medications)	241 (32.7)	57 (37.3)	108 (37.6)	0.099	154 (21.9)	92 (36.9) ^†^	68 (34.7) ^‡^	<0.001
Hospitalization in the past year	73 (9.9)	24 (15.8) ^†^	43 (15.0) ^‡^	0.010	55 (7.9)	45 (18.1) ^†^	32 (16.3) ^‡^	<0.001
Hearing impairment	135 (18.3)	31 (20.3)	88 (30.7) ^‡,^*	<0.001	93 (13.2)	49 (19.7) ^†^	28 (14.4)	0.155
Visual impairment	11 (1.5)	5 (3.3)	13 (4.5) ^‡^	0.005	10 (1.4)	18 (7.2) ^†^	6 (3.1)	0.009
Low chewing ability	262 (35.5)	73 (47.7) ^†^	128 (44.6) ^‡^	0.001	312 (44.4)	138 (55.4) ^†^	111 (56.6) ^‡^	<0.001
ADL disability	5 (0.7)	10 (6.5) ^†^	10 (3.5) ^‡^	0.002	3 (0.4)	13 (5.2) ^†^	3 (1.5)	0.014
IADL disability	41 (5.6)	16 (10.5) ^†^	17 (5.9)	0.407	9 (1.3)	27 (10.8) ^†^	4 (2.0) *	0.004
Falls in the past year	102 (13.9)	39 (25.5) ^†^	54 (18.8) ^‡^	0.008	140 (19.9)	70 (28.1) ^†^	55 (28.2) ^‡^	0.002
Severe mobility limitation	21 (2.8)	31 (20.4) ^†^	27 (9.4) ^‡,^*	<0.001	67 (9.5)	94 (38.2) ^†^	42 (21.5) ^‡,^*	<0.001
Risk of malnutrition (MNA score < 11)	37 (5.0)	26 (17.0) ^†^	40 (14.0) ^‡^	<0.001	42 (6.0)	46 (18.6) ^†^	30 (15.4) ^‡^	<0.001
*Medical* *status*								
Hypertension	391 (53.1)	78 (51.0)	158 (55.1)	0.713	394 (56.0)	150 (60.2)	119 (60.7)	0.146
Diabetes	172 (23.3)	43 (28.1)	81 (28.2)	0.071	121 (17.2)	69 (27.7) ^†^	43 (21.9)	0.006
Urinary incontinence	9 (1.2)	5 (3.3)	8 (2.8)	0.064	27 (3.8)	20 (8.0) ^†^	20 (10.2) ^‡^	<0.001
Cardiovascular disease ^a^	116 (15.7)	27 (17.6)	47 (16.4)	0.692	67 (9.5)	32 (12.9)	15 (7.7)	0.910
Osteoarthritis	80 (10.9)	18 (11.8)	33 (11.5)	0.719	201 (28.6)	77 (30.9)	56 (28.6)	0.744
Osteoporosis	15 (2.0)	9 (5.9) ^†^	14 (4.9) ^‡^	0.010	155 (22.0)	59 (23.7)	63 (32.1) ^‡,^*	0.011
Rheumatoid arthritis	5 (0.7)	1 (0.7)	4 (1.4)	0.307	16 (2.3)	10 (4.0)	14 (7.1) ^‡^	0.002
Chronic obstructive pulmonary disease	10 (1.4)	5 (3.3)	8 (2.8)	0.093	4 (0.6)	1 (0.4)	1 (0.5)	0.815
Thyroid disease	22 (3.0)	1 (0.7)	7 (2.4)	0.311	52 (7.4)	10 (4.0)	14 (7.1)	0.305
Depression	16 (2.2)	5 (3.3)	4 (1.4)	0.690	16 (2.3)	13 (5.2) ^†^	9 (4.6)	0.031
*Psychological and cognitive status*								
EQ-5D index	0.94 ± 0.1	0.87 ± 0.1 ^†^	0.90 ± 0.1 ^‡,^*	<0.001	0.90 ± 0.1	0.81 ± 0.1 ^†^	0.85 ± 0.1 ^‡,^*	<0.001
EQ-VAS	78.4 ± 14.4	69.3 ± 16.2 ^†^	71.7 ± 15.6 ^‡^	<0.001	75.8 ± 16.7	67.6 ± 21.1 ^†^	70.4 ± 17.4 ^‡^	<0.001
SF-12								
Physical health	49.5 ± 7.3	42.7 ± 10.7 ^†^	44.8 ± 9.4 ^‡,^*	<0.001	44.8 ± 9.3	36.5 ± 11.4 ^†^	40.6 ± 10.2 ^‡,^*	<0.001
Mental health	55.1 ± 8.2	54.2 ± 10.2	52.6 ± 9.6 ^‡^	<0.001	53.0 ± 10.0	49.7 ± 12.1 ^†^	48.7 ± 11.5 ^‡^	<0.001
Depressive symptoms (GDS score ≥ 6)	81 (11.0)	40 (26.1) ^†^	65 (22.6) ^‡^	<0.001	157 (22.3)	104 (41.8) ^†^	70 (35.7) ^‡^	<0.001
Cognitive dysfunction (MMSE score < 24)	68 (9.2)	47 (30.7) ^†^	55 (19.2) ^‡,^*	<0.001	135 (19.2)	106 (42.6) ^†^	51 (26.0) ^‡,^*	<0.001
*Social factors*								
Social support	5.4 ± 1.3	5.1 ± 1.7 ^†^	5.3 ± 1.4	0.062	5.4 ± 1.2	5.3 ± 1.4	5.5 ± 1.2	0.780
Poor social capital	47 (6.4)	19 (12.6) ^†^	31 (10.9) ^‡^	0.006	32 (4.6)	27 (11.0) ^†^	15 (7.7)	0.007
Social network								
Low interaction with family	297 (40.3)	47 (30.9) ^†^	113 (39.4)	0.364	258 (36.8)	82 (33.2)	75 (38.3)	0.861
Low interaction with friends	144 (19.5)	44 (28.9) ^†^	66 (23.0)	0.070	89 (12.7)	50 (20.1) ^†^	38 (19.4) ^‡^	0.002
Low interaction with neighbors	273 (37.3)	49 (32.0)	119 (41.8) *	0.423	198 (28.2)	74 (29.7)	57 (29.7)	0.608
Religious activities (none)	380 (51.6)	95 (62.1) ^†^	161 (56.3)	0.052	174 (24.8)	95 (38.2) ^†^	60 (30.6)	0.002
Social activities (none)	167 (22.7)	45 (29.6)	87 (30.3) ^‡^	0.006	157 (22.3)	63 (25.3)	54 (27.6)	0.105

Notes: Values are means (± standard deviations) or frequencies (%). *p*-values were determined using the generalized estimating equation (model-based) due to overlapping participants among three groups. Post hoc analysis was performed using the independent *t*-test for continuous variables and chi-square or Fisher’s exact test for categorical variables (no sarcopenia vs. possible sarcopenia or no sarcopenia vs. sarcopenia), as well as using the generalized estimating equation (possible sarcopenia vs. sarcopenia). * Comparison between possible sarcopenia and sarcopenia. ^†^ Comparison between no sarcopenia and possible sarcopenia. ^‡^ Comparison between no sarcopenia and sarcopenia. Significant difference by post hoc analysis was considered at *p*-value < 0.05. ^a^ Cardiovascular diseases included were myocardial infarction, congestive heart failure, angina, peripheral vascular disease, and cerebrovascular disease. Abbreviations: BMI, body mass index; ASM, appendicular skeletal muscle mass; ADL, activities of daily living; IADL, instrumental activities of daily living; MNA, Mini-Nutritional Assessment; EQ-5D, EuroQol-5 dimension; EQ-VAS, EuroQol Visual Analog Scale; SF-12, 12-item short-form health survey; GDS, Geriatric Depression Scale; MMSE, Mini-Mental State Exam.

**Table 2:** Adjusted odds ratios of variables for predicting possible sarcopenia compared with sarcopenia in men.

Independent Variable	Model 1		Model 2		Model 3		Model 4	
	OR (95% CI)	*p*- Value	OR (95% CI)	*p*- Value	OR (95% CI)	*p*- Value	OR (95% CI)	*p*- Value
*Sociodemographic and lifestyle factors*								
Low education level, <7 years	2.03 (1.32–3.10)	0.001	2.03 (1.32–3.13)	0.001	1.97 (1.26–3.08)	0.003	1.78 (1.09–2.90)	0.021
*Anthropometric measurements and physical performance*								
Timed Up and Go test, s			1.11 (1.04–1.19)	0.003	1.09 (1.01–1.19)	0.037	1.09 (1.00–1.19)	0.043
*Medical and health status*								
Hearing impairment					0.48 (0.29–0.79)	0.004	0.54 (0.32–0.93)	0.025
IADL disability					1.24 (0.53–2.91)	0.620	1.27 (0.51–3.15)	0.606
Severe mobility limitation					1.48 (0.74–2.97)	0.268	1.74 (0.75–4.02)	0.197
*Psychological, cognitive, and social factors*								
EQ-5D index							0.78 (0.07–8.26)	0.838
SF-12 (physical health)							1.00 (0.97–1.03)	0.897
SF-12 (mental health)							1.04 (1.00–1.07)	0.037
Cognitive dysfunction (MMSE score < 24)							1.31 (0.78–2.20)	0.304
Social network (with family)							0.81 (0.52–1.27)	0.363
Social network (with neighbor)							0.77 (0.49–1.23)	0.282

*p*-values were determined using the generalized estimating equation (model-based) due to overlapping participants in the possible sarcopenia and sarcopenia groups. Variables with a *p*-value of <0.1 in the univariate analysis of possible sarcopenia and sarcopenia groups were entered into the model. Abbreviations: OR, odds ratio; CI, confidence interval; BMI, body mass index; ADL, activities of daily living; EQ-5D, EuroQol-5 dimension; SF-12, 12-item short-form health survey; MMSE, Mini-Mental State Examination.

**Table 3:** Adjusted odds ratios of variables for predicting possible sarcopenia compared with sarcopenia in women.

Independent Variable	Model 1		Model 2		Model 3		Model 4	
	OR (95% CI)	*p*- Value	OR (95% CI)	*p*- Value	OR (95% CI)	*p*- Value	OR (95% CI)	*p*- Value
*Sociodemographic and lifestyle factors*								
Age, years	1.02 (0.97–1.08)	0.373	0.99 (0.93–1.05)	0.659	0.99 (0.93–1.05)	0.687	0.98 (0.93–1.05)	0.613
Low education level, <7 years	2.27 (1.52–3.40)	<0.001	2.13 (1.41–3.24)	<0.001	2.04 (1.33–3.13)	0.001	1.91 (1.17–3.14)	0.010
Marital status (without partner)	1.42 (0.94–2.14)	0.096	1.46 (0.96–2.23)	0.079	1.45 (0.94–2.24)	0.093	1.50 (0.96–2.33)	0.073
Low physical activity	1.36 (0.82–2.27)	0.239	0.97 (0.56–1.69)	0.920	0.86 (0.48–1.53)	0.595	0.84 (0.46–1.54)	0.570
*Anthropometric measurements and physical performance*								
BMI, kg/m^2^			1.06 (0.95–1.19)	0.277	1.05 (0.94–1.17)	0.415	1.04 (0.92–1.16)	0.543
Waist circumference, cm			1.01 (0.98–1.05)	0.480	1.02 (0.98–1.06)	0.366	1.02 (0.98–1.06)	0.398
Timed Up and Go test, s			1.14 (1.05–1.23)	0.002	1.10 (1.01–1.20)	0.038	1.08 (0.99–1.19)	0.098
*Medical and health status*								
Visual impairment					2.19 (0.78–6.17)	0.139	1.85 (0.64–5.38)	0.259
ADL disability					1.54 (0.35–6.83)	0.569	1.54 (0.33–7.14)	0.583
IADL disability					3.88 (1.19–12.69)	0.025	4.61 (1.21–17.50)	0.025
Severe mobility limitation					1.06 (0.64–1.77)	0.816	0.98 (0.56–1.72)	0.943
Cardiovascular diseases ^a^					1.37 (0.66–2.84)	0.392	1.35 (0.63–2.85)	0.440
Osteoporosis					0.77 (0.49–1.21)	0.254	0.74 (0.46–1.19)	0.213
*Psychological, cognitive, and social factors*								
EQ-5D index							1.26 (0.17–9.46)	0.821
SF-12 (physical health)							0.98 (0.96–1.01)	0.161
Cognitive dysfunction (MMSE < 24)							1.15 (0.68–1.94)	0.611
Religious activities (none)							0.98 (0.61–1.58)	0.943

*p*-values were determined using the generalized estimating equation (model-based) due to overlapping participants in the possible sarcopenia and sarcopenia groups. Variables with a *p*-value of <0.1 in the univariate analysis of possible sarcopenia and sarcopenia groups were entered into the model. ^a^ Cardiovascular diseases included were myocardial infarction, congestive heart failure, angina, peripheral vascular disease, and cerebrovascular disease. Abbreviations: OR, odds ratio; CI, confidence interval; BMI, body mass index; ADL, activities of daily living; MNA, Mini-Nutritional Assessment; EQ-5D, EuroQol-5 dimension; EQ-VAS, EuroQol Visual Analog Scale; SF-12, 12-item short-form health survey; GDS, Geriatric Depression Scale; MMSE, Mini-Mental State Examination.Predictors of possible sarcopenia and sarcopenia compared with the no sarcopenia are shown in Supplementary Materials: Tables S1 and S2.

## Data Availability

The data that support the findings of this study are available on request from the corresponding author. The data are not publicly available, due to privacy or ethical restrictions.

## Supplementary Materials

The following supporting information can be downloaded at: https://www.mdpi.com/article/10.3390/ijerph19074312/s1, Table S1: Adjusted odds ratios of variables for predicting possible sarcopenia compared with no sarcopenia; Table S2: Adjusted odds ratios of variables for predicting sarcopenia compared with no sarcopenia.
