# Supplementary material for: Differences in Characteristics between Older Adults Meeting Criteria for Sarcopenia and Possible Sarcopenia: From Research to Primary Care
Source: Int J Environ Res Public Health. 2022 Apr 4;19(7):4312. doi: 10.3390/ijerph19074312 (PMC8998728; doi:10.3390/ijerph19074312)
Supplement: Supplementary file 1 [file ijerph-19-04312-s001.zip › ijerph-1592587-supplementary.pdf]

## Supplementary Materials:

**Table S1.** Adjusted odds ratios of variables for predicting possible sarcopenia compared with no sarcopenia.

| **Men** | No sarcopenia to Possible sarcopenia | |
|---|---|---|
| | OR (95% CI) | *p*-value |
| Age, years | 1.18 (1.10-1.27) | <0.001 |
| BMI, kg/m² | 0.53 (0.44-0.64) | <0.001 |
| Waist circumference, cm | 1.11 (1.04-1.17) | 0.001 |
| Timed Up and Go test, sec | 1.59 (1.39-1.82) | <0.001 |
| Hospitalization in past year | 3.02 (1.45-6.26) | 0.003 |
| SF-12 (physical health) | 0.96 (0.93-1.00) | 0.039 |
| Social networks (with family) | 0.38 (0.22-0.68) | 0.001 |
| Religious (none) | 2.18 (1.25-3.82) | 0.006 |
| **Women** | No sarcopenia to Possible sarcopenia | |
| | OR (95% CI) | *p*-value |
| BMI, kg/m² | 0.68 (0.60-0.78) | <0.001 |
| Waist circumference, cm | 1.04 (1.00-1.09) | 0.035 |
| Timed Up and Go test, sec | 1.42 (1.28-1.58) | <0.001 |
| ADL disability | 19.59 (2.03-189.42) | 0.010 |
| Diabetes | 1.64 (1.01-2.67) | 0.045 |
| SF-12 (physical health) | 0.97 (0.94-0.99) | 0.006 |
| Religious activities (none) | 1.63 (1.02-2.59) | 0.039 |

*p*-value was determined using the multiple logistic regression model. Variables with a *p*-value of <0.1 in the univariate analysis between normal (no sarcopenia) and possible sarcopenia were entered into the model. *Abbreviations:* OR, odds ratio; CI, confidence interval; BMI, body mass index; ADL, activities of daily living; SF-12, 12-item short form health survey.

**Table S2.** Adjusted odds ratios of variables for predicting sarcopenia compared with no sarcopenia.

| **Men** | No sarcopenia to Sarcopenia | |
|---|---|---|
| | OR (95% CI) | *p*-value |
| Age, years | 1.19 (1.13-1.25) | <0.001 |
| BMI, kg/m² | 0.51 (0.44-0.59) | <0.001 |
| Waist circumference, cm | 1.14 (1.10-1.19) | <0.001 |
| Timed Up and Go test, sec | 1.44 (1.30-1.59) | <0.001 |
| SF-12 (physical health) | 0.97 (0.94-1.00) | 0.047 |
| **Women** | No sarcopenia to Sarcopenia | |
| | OR (95% CI) | *p*-value |
| BMI, kg/m² | 0.64 (0.56-0.73) | <0.001 |
| Timed Up and Go test, sec | 1.29 (1.17-1.43) | <0.001 |
| Rhematoid arthritis | 2.50 (1.00-6.24) | 0.049 |
| SF-12 (mental health) | 0.97 (0.95-0.99) | 0.003 |

*p*-value was determined using the multiple logistic regression model. Variables with a *p*-value of <0.1 in the univariate analysis were entered into the model between normal (no sarcopenia) and sarcopenia. *Abbreviations:* OR, odds ratio; CI, confidence interval; BMI, body mass index; ADL, activities of daily living; SF-12, 12-item short form health survey.
